# Prevention of migraine with monoclonal antibodies against CGRP or the CGRP receptor: Addition to the S1 guideline: Therapy of migraine attacks and prevention of migraine. Recommendations of the Germany Society of Neurology and the German Migraine and Headache Society

**DOI:** 10.1186/s42466-020-00057-1

**Published:** 2020-04-13

**Authors:** Hans-Christoph Diener, Stefanie Förderreuther, Charly Gaul, Florian Giese, Till Hamann, Dagny Holle-Lee, Tim P. Jürgens, Katharina Kamm, Torsten Kraya, Christian Lampl, Arne May, Uwe Reuter, Armin Scheffler, Peer Tfelt-Hansen

**Affiliations:** 1 Medizinische Fakultät der Universität Duisburg-Essen, Institut für Medizinische Informatik, Biometrie und Epidemiologie (IMIBE), Hufelandstr. 26, 45147 Essen, Germany; 2 Neurologische Klinik, Ludwig-Maximilians-Universität München, Klinikum Großhadern, Marchioninistr. 15, 81377 Munich, Germany; 3 Migräne- und Kopfschmerzklinik Königstein, Ölmühlweg 31, 61462 Königstein im Taunus, Germany; 4 Klinik und Poliklinik für Neurologie, Universitätsklinikum Halle, Gütchenstr. 14, 06108 Halle, Germany; 5 Klinik und Poliklinik für Neurologie und Kopfschmerzzentrum Nord-Ost, Universitätsmedizin Rostock, Gehlsheimer Str. 20, 18147 Rostock, Germany; 6 Klinik für Neurologie und Westdeutsches Kopfschmerzzentrum Universitätsklinikum Essen, Hufelandstr. 55, 45147 Essen, Germany; 7 Neurologische Klinik, Ludwig-Maximilians-Universität München, Klinikum Großhadern, Marchioninistr. 15, 81377 Munich, Germany; 8 Klinik für Neurologie, Klinikum St. Georg Leipzig, Delitzscher Str. 141, 04129 Leipzig, Germany; 9 Akutgeriatrie und Remobilisation, Kopfschmerzzentrum Seilerstätte, Ordensklinikum Linz Barmherzige Schwestern, Seilerstätte 4, A-4010 Linz, Austria; 10 Institut für Systemische Neurowissenschaften, Universitätsklinikum Hamburg-Eppendorf (UKE), Gebäude W34, 3. Stock, Martinistraße 52, 20246 Hamburg, Germany; 11 Kopfschmerzzentrum, Charité Universitätsmedizin Berlin, Charitéplatz 1, 10117 Berlin, Germany; 12 Klinik für Neurologie, Universitätsklinikum Essen, Hufelandstr. 55, 45147 Essen, Germany; 13 Department of Neurology, Danish Headache Center, Rigshospitalet-Glostrup Hospital, University Hospital of Copenhagen, Glostrup Hospital, Ringvejen, DK-2600 Glostrup, Denmark

**Keywords:** Episodic migraine, Chronic migraine, Migraine prevention, CGRP, Monoclonal antibodies, Guideline

## Abstract

Monoclonal antibodies against the calcitonin gene-related peptide (CGRP) receptor (Erenumab) or against CGRP (Eptinezumab, Fremanezumab, Galcanezumab) are new substances for the preventive treatment of migraine. They represent an extension of the therapeutic options, which already exist in migraine prevention. In randomized, placebo-controlled studies, the efficacy and good tolerability of these specific substances have been demonstrated in patients with episodic and chronic migraine. The following treatment recommendation presents a summary of the pivotal studies. Recommendations are provided for the targeted selection of patients as well as for the evaluation of therapeutic success and the duration of treatment. Finally, possible restrictions on the use of this new substance group are discussed.

This guideline is an abridged and translated version of the guideline published by Diener H-C, May A et al., Prevention of migraine with monoclonal antibodies against CGRP or the CGRP receptor, Supplement to S1 Guideline Therapy of Migraine Attack and Prevention of Migraine, 2019, Deutsche Gesellschaft für Neurologie (eds.), Guidelines for Diagnostics and Therapy in Neurology. A complete version of this guideline can be found on the website of the Deutsche Gesellschaft für Neurologie (www.dgn.org/leitlinien) and the AWMF (Arbeitsgemeinschaft wissenschaftlicher Medizinischer Gesellschaften).

This guideline has been approved by the German Neurological Society (DGN) and the German Migraine and Headache Society (GMHS) and was reviewed by the two societies.

## Introduction

This guideline is an abridged and translated version of the guideline published by Diener H-C, May A et al., Prevention of migraine with monoclonal antibodies against CGRP or the CGRP receptor, Supplement to S1 Guideline Therapy of Migraine Attack and Prevention of Migraine, 2019, Deutsche Gesellschaft für Neurologie (eds.), Guidelines for Diagnostics and Therapy in Neurology [1]. A complete version of this guideline can be found on the website of the Deutsche Gesellschaft für Neurologie (www.dgn.org/leitlinien) and the AWMF (Arbeitsgemeinschaft wissenschaftlicher Medizinischer Gesellschaften). The original guidelines was published in August 2019 and the guidance is valid until 1 September 2022.

Recently, three monoclonal antibodies against CGRP and the CGRP receptor have become available for the medicinal prophylaxis of migraine. A further one (Eptinezumab) was approved in the USA. Therefore, the existing guideline for the treatment of migraine attack and prophylaxis of migraine of DGN and DMKG had to be updated. This guideline deals with the therapeutic use of antibodies against CGRP or the CGRP receptor for the prophylaxis of episodic or chronic migraine. The patient group for whom this guidance is most relevant are those patients with migraine in whom previous drug therapies have been ineffective, were not tolerated or are contraindicated. The scope of application of the guideline covers outpatient, day-care and inpatient care and the recommendations of the guideline are aimed at neurologists and pain therapists who treat patients with therapy-refractory migraine.

Patients with frequent or severe migraine attacks require non-drug and/or drug migraine prevention in addition to effective treatment of the acute migraine attack [1]. Until now, according to the Guidelines of the German Society of Neurology and the German Migraine and Headache Society, the beta-receptor blockers propranolol, metoprolol and bisoprolol, the calcium antagonist flunarizine, the anticonvulsants valproic acid and topiramate as well as the tricyclic antidepressant amitriptyline have been available for this purpose with a high degree of evidence [1]. According to a decision of the German Federal Joint Committee, specialists in neurology or psychiatry (https://www.g-ba.de/downloads/39-261-3911/2019-08-06_AM-RL-VI-SN_Valproinsäure-Migräneprophylaxe.pdf) may prescribe valproic acid for the treatment of migraine. The need for consistent, safe contraception must be explained to the patient in writing. Topiramate and onabotulinumtoxinA are effective in chronic migraine. The drugs used so far for migraine prevention have comparable efficacy. According to the current recommendations of the International Headache Society, the 50% response rate is used as the target criterion [2]. The 50% response rate describes the percentage of migraine patients in whom a reduction of migraine days/month by ≥50% compared to baseline achieved after 3 months of therapy. In patients who did not achieve the 50% response rate, the individual drug groups often had to be used consecutively and sometimes additively to find an effective and tolerable therapy.

Drugs for migraine prevention are effective in many patients. However, a major problem of most of the migraine preventive drugs available to date is adverse events. This explains why adherence and persistence are low [3]. There was a need to develop new drugs for migraine prevention with a more favorable side effect profile.

## Background into calcitonin gene-related peptides (CGRP) and migraine

In 1991, Goadsby and Edvinsson identified the important role of CGRP in the pathophysiology of migraine [4, 5]. They systematically investigated neuropeptides in blood samples from the jugular vein during acute migraine attacks. They found that CGRP was released during migraine attacks and that CGRP concentrations decreased when the attack was successfully treated with subcutaneous sumatriptan. Thereafter, CGRP was detected in the human trigeminal ganglion [6] and in the walls of cerebral arteries [5, 7]. In the following years, Goadsby and Edvinsson identified CGRP receptors in the walls of cerebral vessels and arteries of the dura, in the trigeminal-vascular system and in central pain-conducting structures [8]. CGRP is a potent vasodilator [9]. The final proof that intervention in the CGRP circuit is effective against migraine attacks was a placebo-controlled study by Olesen and Diener in which a CGRP antagonist was significantly effective in treating migraine attacks [10].

**Fig. 1:**
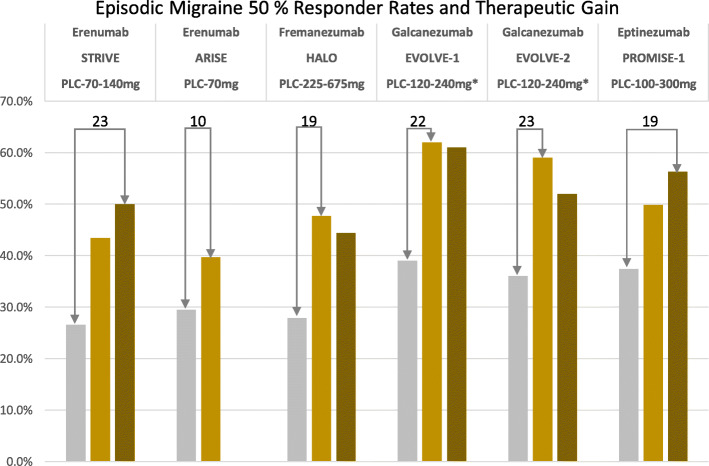
50% responder rates for the prevention of episodic migraine. The numbers on top of the columns reflect therapeutic gain (verum minis placebo)

**Fig. 2:**
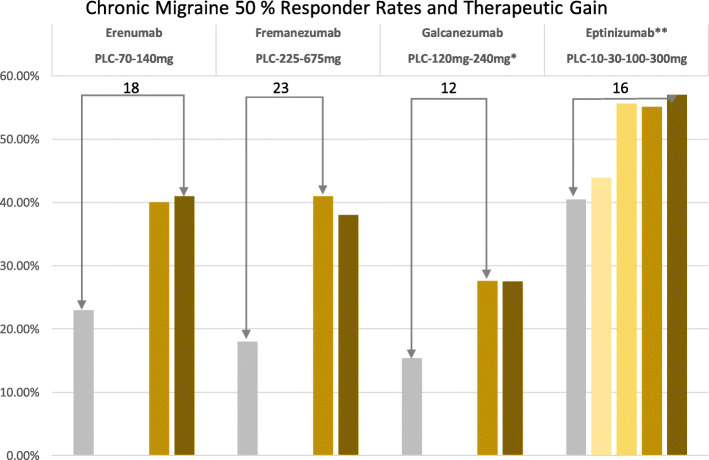
50% responder rates for the prevention of chronic migraine. The numbers on top of the columns reflect therapeutic gain (verum minis placebo)

Four monoclonal antibodies - Eptinezumab, Erenumab, Fremanezumab, and Galcanezumab - have undergone extensive clinical trials in episodic and chronic migraine and demonstrated superiority over placebo [11]. The results for the prevention of episodic and chronic migraine as a therapeutic benefit (50% responder rate verum minus placebo) are shown in Figs. 1 and 2. Monoclonal antibodies have a molecular weight of around 150 kDa and are unable to cross the intact blood-brain barrier. Therefore, they are called “large molecules” in contrast to conventional pharmaceuticals and the “-gepants” [12], which represent “small molecules”. Due to the manufacturing process, fully human, recombinant antibodies (ending “umab”) are distinguished from humanized antibodies, which still contain murine components (ending “zumab”). Fully human and humanized monoclonal antibodies are highly specific and lead to the formation of autoantibodies only to a minimal extent. Due to their biological properties, antibodies against the ligand CGRP or the CGRP receptor itself have a favorable side effect profile. This is due to the size of the antibodies, as they do not cross the blood-brain barrier to a relevant extent and do not have central nervous system side effects. Due to their degradation to amino acids, they do not interact with other drugs by bypassing hepatic and renal elimination steps. Monoclonal antibodies have to be administered either subcutaneously or intravenously, with the corresponding dosing intervals depending on the half-life and dose-ranging between 4 weeks and 3 months for Fremanezumab and Eptinezumab.

## Methods of guideline development

Research, selection of proven scientific evidence: First, seven questions were formulated on the use of monoclonal antibodies against CGRP or the CGRP receptor and a systematic literature search was conducted for these questions. The terms CGRP, CGRP antibody, monoclonal antibody against CGRP, CGRP-receptor antagonist, Erenumab, Fremanezumab, Galcanezumab, Eptinezumab, episodic migraine, chronic migraine, safety, tolerability, adverse events, medication overuse headache were used. The literature review covered the period from January 2015 until mid-August 2019. Selection of evidence: The relevant literature was selected by the authors who formulated the respective section of the guideline.

The guideline is an addition to the S1 DGN–DMKG guideline: Therapy of migraine attacks and prevention of migraine (AWMF-Registry number 030/057).

The guideline was released on 30 August 2019 and is valid until 1 September 2022.

These are joint recommendations of the Germany Society of Neurology and the German Migraine and Headache Society. Both societies reviewed the guideline.

The external review of the guideline was carried out by three members of the DGN Guidelines Commission and additionally by two independent external reviewers of the DGN.

## Recommendations

**Fig. 3:**
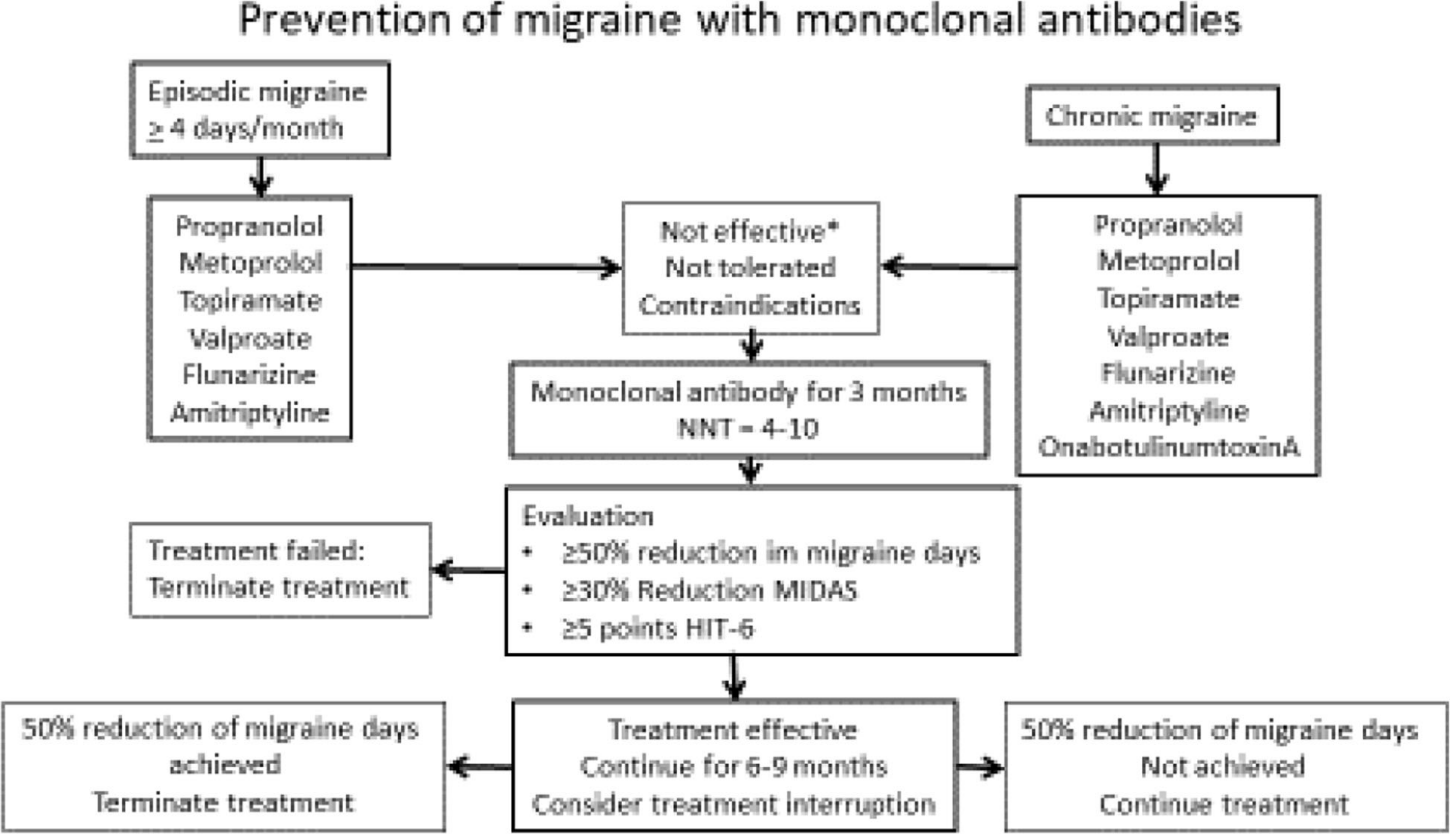
Flow diagram for migraine prevention with monoclonal antibodies

The treatment algorithm for prevention of migraine with monoclobal antibodies can be seen in Fig. 3. The seven different recommendations are explained below.

## 1. Are monoclonal antibodies (MOAB) against CGRP or the CGRP receptor effective for the prevention of episodic migraine?

Monoclonal antibodies against CGRP (Eptinezumab, Fremanezumab, and Galcanezumab) or the CGRP receptor (Erenumab) are superior to placebo treatment in the preventive treatment of episodic migraine. The reduction of migraine days per month in episodic migraine ranges between 2.9 and 4.7 days. The 50% responder rate after 3–6 months is between 30 and 62%. The 50% responder rate for placebo is between 17 and 38%. The effectiveness can be evaluated within 4–8 weeks. A direct comparison of the monoclonal antibodies with each other has not been performed, nor is a comparison with the traditional migraine preventive drugs available so far.

## 2. Are monoclonal antibodies against CGRP or the CGRP receptor effective in the prevention of chronic migraine?

Monoclonal antibodies against CGRP (Eptinezumab, Fremanezumab, and Galcanezumab) or the CGRP receptor (Erenumab) are superior to placebo treatment in the prevention of chronic migraine. The reduction of migraine days per month for chronic migraine is between 4.3 and 6.6 days. The 50% response rate after 3 months ranges between 27 and 57%. The 50% response rate for placebo is between 15 and 40%. Efficacy has also been shown for patients with chronic migraine and medication overuse. A direct comparison of the monoclonal antibodies with each other was not performed, nor is a comparison with traditional migraine preventive drugs available to date.

## 3. Which patients should receive a monoclonal antibody for migraine prevention?

Monoclonal antibodies are approved in Germany for the treatment of migraine with at least four migraine days per month. According to the decision of the German Federal Joint Committee (GBA), a prescription is possible in patients with episodic migraine if at least 5 substances from the 4 available, approved pharmacological groups such as beta-blockers (Metoprolol or Propranolol), Flunarizine, Topiramate, valproic acid or amitriptyline were not effective, not tolerated or if there are contraindications or warnings against their use. Regarding patients with chronic migraine, it is recommended that they have not additionally responded to therapy with OnabotulinumtoxinA.

## 4. How is the therapy success evaluated?

In episodic and chronic migraine, treatment success is defined as a reduction in the average monthly headache days by 50% or more compared to pre-treatment for a period of at least 3 months (diary documentation is recommended) [13].

Alternative clinically acceptable criteria are significant improvements in validated, migraine-specific, patient-related outcome measures such as.

30% reduction of the MIDAS [14] score for those with baseline values above 20.

Reduction of the score in the 6-point headache impact test (HIT-6) [15] by at least 5 points.

## 5. How long should preventive therapy be performed?

The therapy should initially be carried out for 3 months. If there is no satisfying therapy effect, the therapy will be terminated. If the therapy is effective, interruption of therapy should be considered after 6–9 months to check whether the therapy is still necessary.

## 6. What are the contraindications and warnings for the use of monoclonal antibodies?

Monoclonal antibodies against CGRP or the CGRP receptor should not be used in pregnant women and during lactation. They should not be used in women who have no or insufficient contraception. Furthermore, as a precaution, they should not be used in patients with coronary heart disease, ischemic stroke, subarachnoidal hemorrhage or peripheral arterial occlusive disease. For children and adolescents, there is no information on tolerability and safety to date. Monoclonal antibodies should not be used in patients with inflammatory bowel disease, COPD, pulmonary hypertension, Raynaud’s syndrome, wound healing disorders or transplant recipients until further notice. Since the available studies have so far only included patients without relevant previous diseases, patients with chronic diseases should be treated with caution.

## 7. Does it make sense to switch to a CGRP receptor antagonist if therapy with a CGRP antagonist does not respond and vice versa?

There are no data on this question from the randomized studies or registries. The attempt to change therapy seems justified.

## Data Availability

Not applicable.
